# Supplementary material for: Mechanism of hormone and allosteric agonist mediated activation of follicle stimulating hormone receptor
Source: Nat Commun. 2023 Jan 31;14:519. doi: 10.1038/s41467-023-36170-3 (PMC9889800; doi:10.1038/s41467-023-36170-3)
Supplement: Supplementary file 1 — Supplementary Information [file 41467_2023_36170_MOESM1_ESM.pdf]

Supplementary Information

Mechanism of hormone and allosteric agonist mediated activation of follicle stimulating hormone receptor

Supplementary Figure 1. Cryo-EM images and single-particle reconstruction of the FSH-FSHR-Gs complex

Supplementary Figure 2. The characteristic of full-length FSHR structure and cryo-EM density maps with all transmembrane helices, H8, P10 fragment and the extended hinge loop of the active FSHR structure

Supplementary Figure 3. Cryo-EM density maps of the cholesterol and lipid in the FSH-FSHR-Gs complex

Supplementary Figure 4. Cryo-EM images and single-particle reconstruction of the inactive FSHR

Supplementary Figure 5. Structural comparison of FSHR structures with the crystal structures of FSH-FSHR_HB and FSH-FSHR_ECD, and the cryo-EM structures of LHCGR and TSHR

Supplementary Figure 6. Sequence alignment of FSHR, LHCGR and TSHR

Supplementary Figure 7. Allosteric agonists interact with glycoprotein hormone receptors

Supplementary Figure 8. The binding free energy between ML-109 and the receptor calculated by MMGBSA

Supplementary Figure 9. Distribution of diseases associated mutations on the FSHR, LHCGR and TSHR structures

Supplementary Figure 10. The representative flow cytometry data for the detection of receptor surface expression

Supplementary Table 1. Cryo-EM data collection, model refinement, and validation statistics

Supplementary Table 2. FSH and Cpd-21f-induced activation on wild type and FSHR with site-directed mutations

Supplementary Table 3. Allosteric agonists-induced activation on wild type and mutated FSHR

Supplementary Table 4. Agonists-induced activation on wild type LHCGR and TSHR

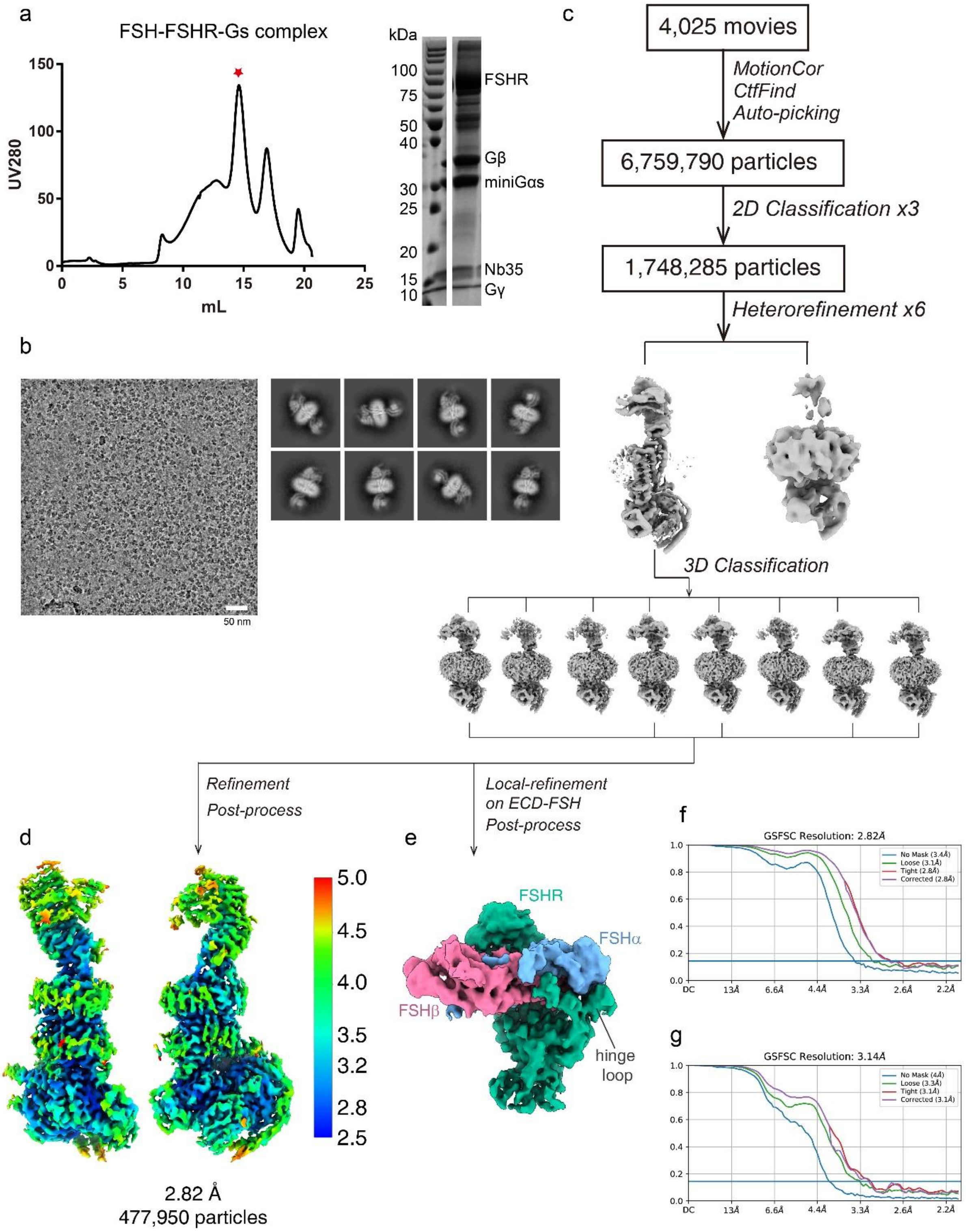

Supplementary Figure 1 | Cryo-EM images and single-particle reconstruction of the FSH-FSHR-Gs complex. a, Size-exclusion chromatography profile and SDS-PAGE of the FSH-FSHR-Gs complex. Red star indicates the peak of the complex. Source data are provided with this paper. The data collection was performed once. **b-e,** Cryo-EM micrograph (**b**), 4025 movies were collected, reference-free 2D class averages (**b**), flowchart of cryo-EM data analysis of the FSH-FSHR-Gs complex (**c**), and cryo-EM map of the FSH-FSHR-Gs complex colored by local

resolutions from 2.5 Å (blue) to 5.0 Å (red) (**d**), and cryo-EM map of the FSH-FSHR ECD subcomplex from local refinement (**e**). **f-g,** The "Gold-standard" Fourier shell correlation (FSC) curves indicate that the overall resolution of the FSH-FSHR-Gs complex is 2.82 Å (**f**), and the local resolution of the FSH-FSHR ECD subcomplex is 3.14 Å (**g**).

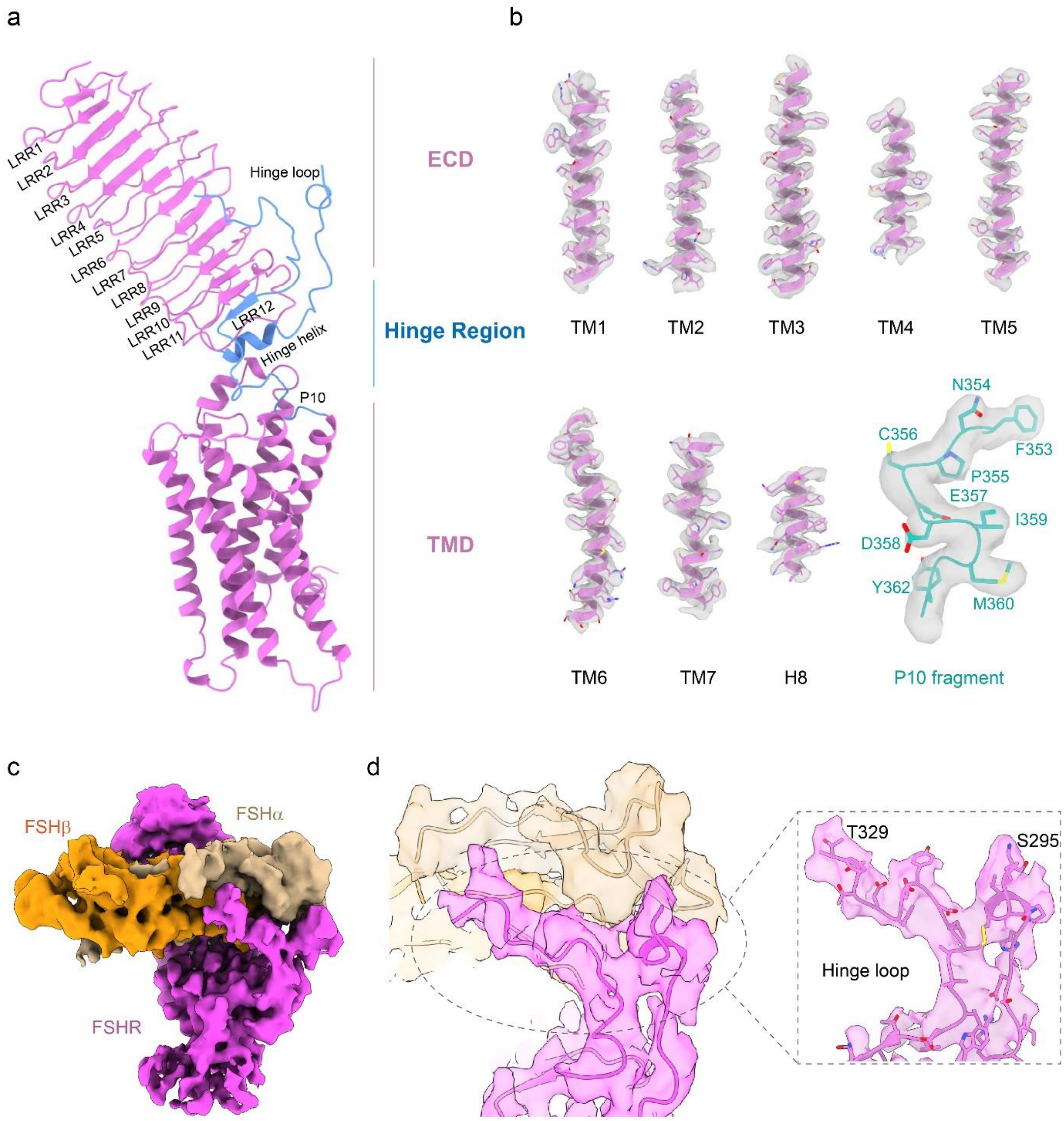

Supplementary Figure 2 | The characteristic of full-length FSHR structure and cryo-EM density maps with all transmembrane helices, H8, P10 fragment and the extended hinge loop of the active FSHR structure. a, Structural components of full-length FSHR structure. **b,** Representative EM densities (gray surface) of all transmembrane helices, H8, P10 fragment. **c, d,** The EM density of FSH-FSHR ECD subcomplex (**c**) and the extended hinge loop from FSHR hinge region (**d**).

a

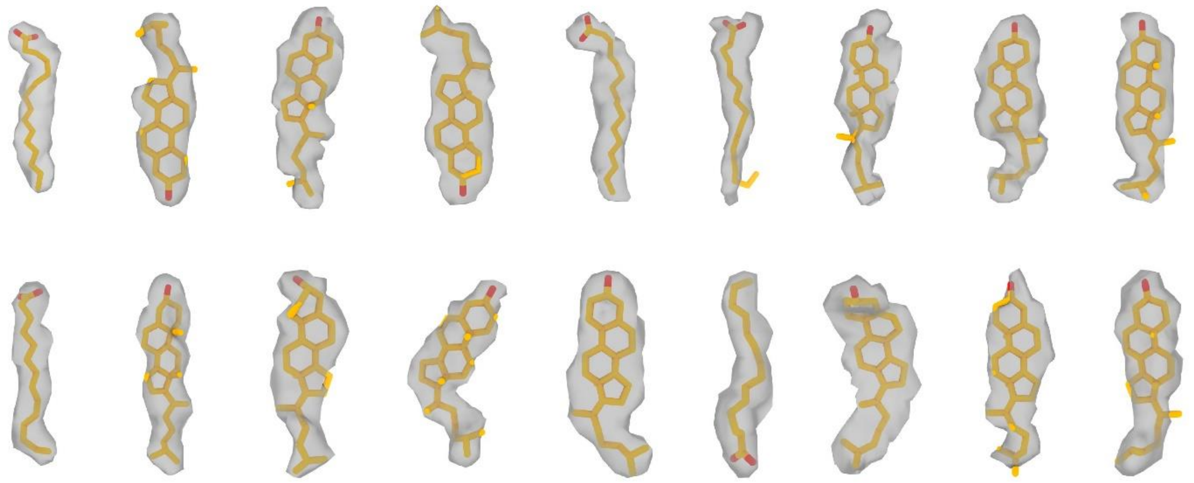

b

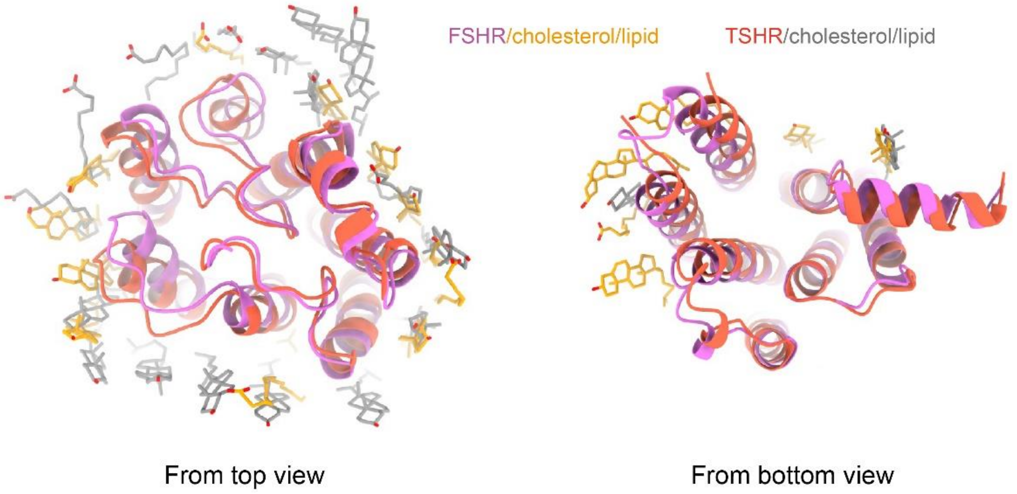

Supplementary Figure 3 | Cryo-EM density maps of the cholesterol and lipid in the FSH-FSHR-Gs complex. a, Cholesterol and lipid density maps in the FSH-FSHR-Gs complex. **b,** Structural comparison of FSH-FSHR-Gs and TSH-TSHR-Gs complexes, cholesterol and lipid in the FSH-FSHR-Gs complex are shown in yellow, and in the TSH-TSHR-Gs complex are shown in grey.

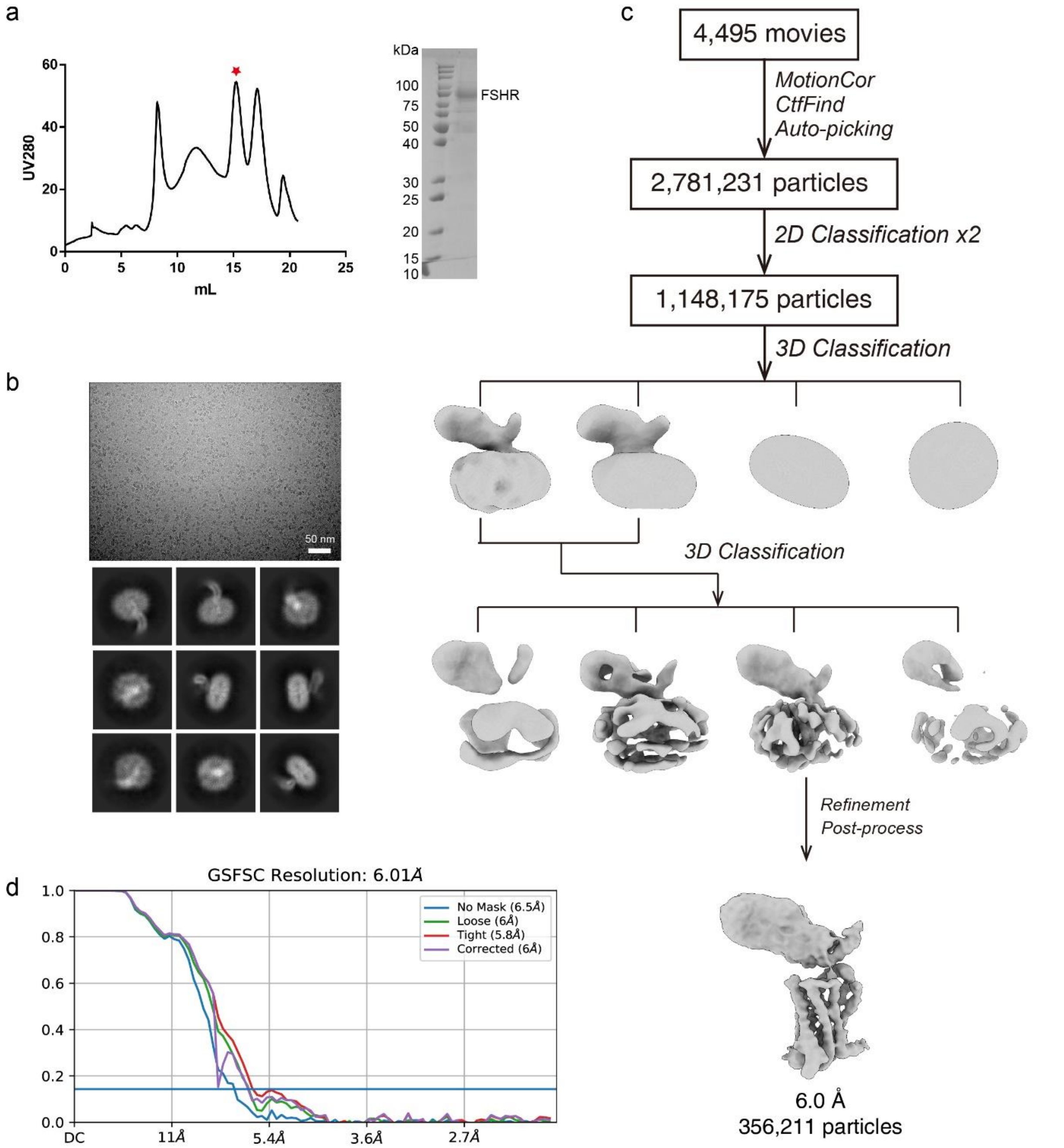

Supplementary Figure 4 | Cryo-EM images and single-particle reconstruction of the inactive FSHR. a, Size-exclusion chromatography profile and SDS-PAGE of the inactive FSHR protein. Red star indicates the peak of the FSHR protein. Source data are provided with this paper. The data collection was performed once. **b, c,** Cryo-EM micrograph (**b**), 4495 movies were collected, reference-free 2D class averages (**b**), flowchart of cryo-EM data analysis of the inactive FSHR protein (**c**), and cryo-EM map of the inactive FSHR protein (**c**). **d,** The “Gold-standard” Fourier shell correlation (FSC) curves indicate that the overall resolution of the inactive FSHR is 6.01 Å.

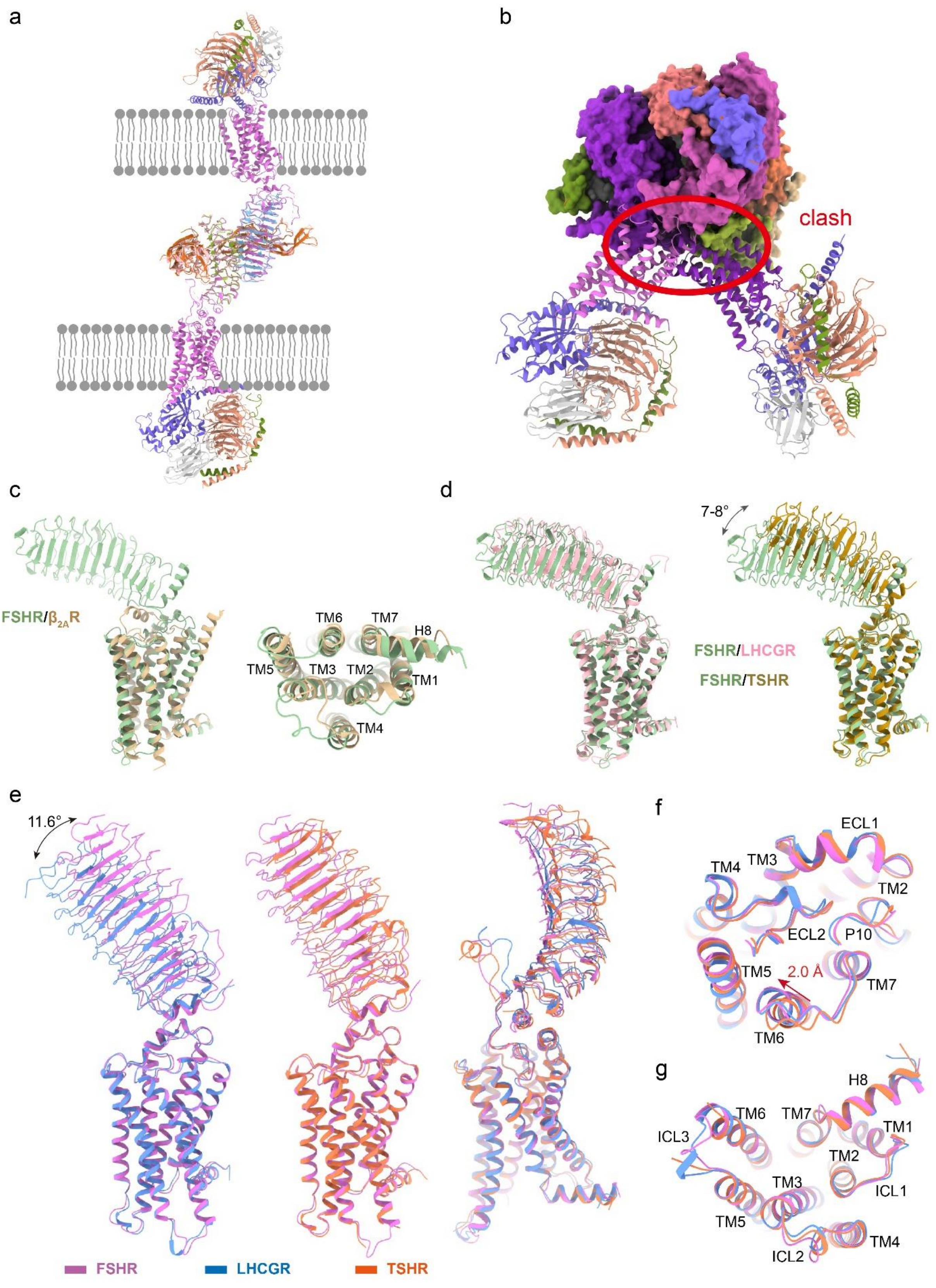

Supplementary Figure 5 | Structural comparison of FSHR structures with the crystal structures of FSH-FSHR_HB and FSH-FSHR_ECD, and the cryo-EM structures of LHCGR and TSHR. a, Superposition of the FSH-FSHR-Gs complex with the FSH-FSHR-HB dimer (PDB code: 1XWD) shows that the two TMDs of FSHR are located at two separately membrane layers

instead of one same membrane layer. Full-length FSHR structures are shown in purple. **b,** Superposition of the FSH-FSHR-Gs complex with the FSH-FSHR-ECD trimer (PDB code: 4AY9) shows that the adjacent two TMDs of FSHR clash with each other. **c,** Conformational comparison of the inactive FSHR with the inactive β_{2A}R (PDB code: 5JQH). **d,** Conformational comparison of the inactive FSHR with the inactive LHCGR (PDB code: 7fij, left panel) and the inactive TSHR (PDB code: 7XW7, right panel). **e-g,** Conformational comparison of the active FSHR with the active LHCGR (PDB code: 7fih, left panel) and the active TSHR (PDB code: 7XW5, right panel). The ECD shift angle between LHCGR and FSHR was measured at Cα atoms of D84 in LHCGR, D81 and Y271 in FSHR.

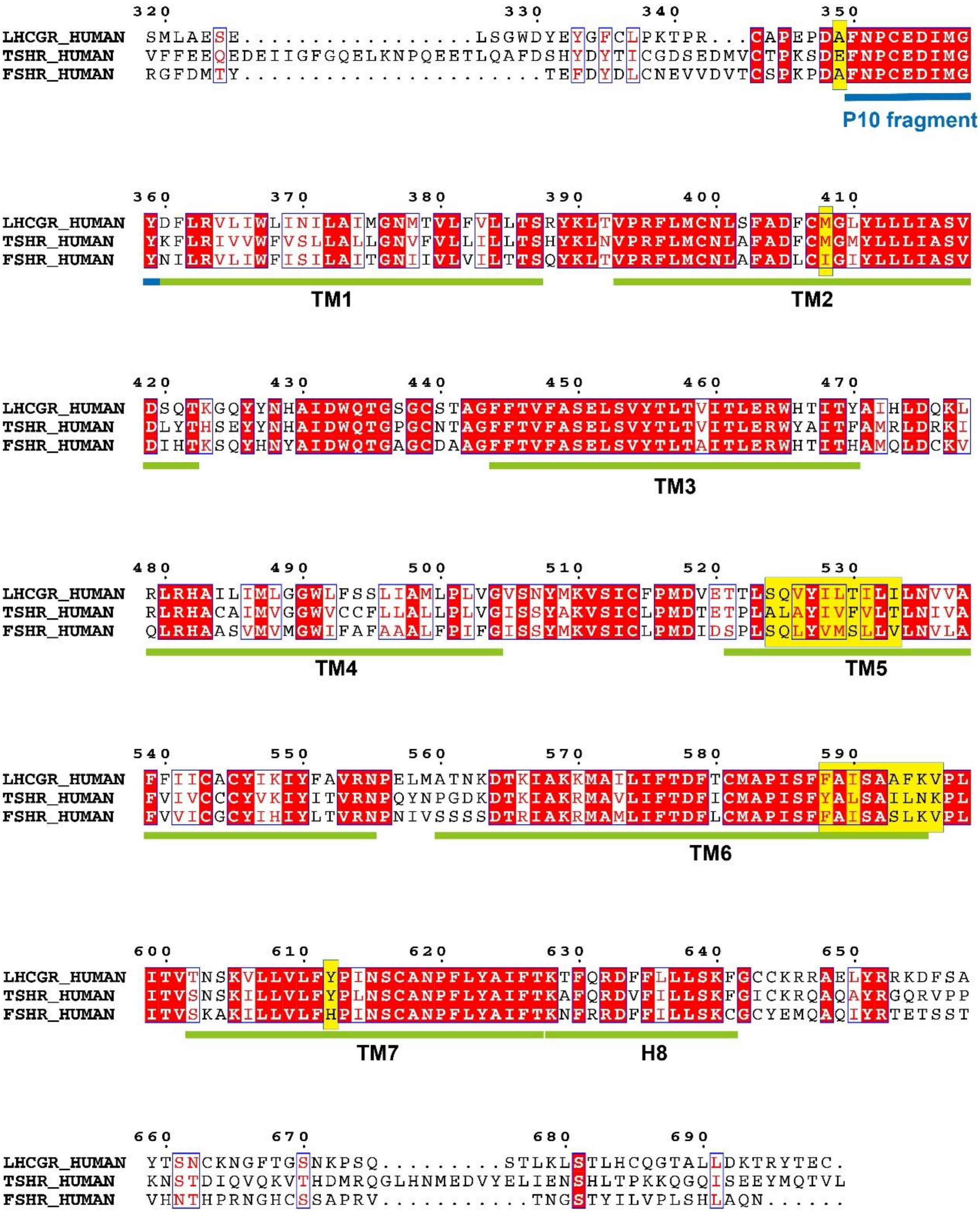

Supplementary Figure 6 | Sequence alignment of FSHR, LHCGR and TSHR. Sequences alignment of human FSHR, LHCGR and TSHR in the region of the hinge region C-terminus and TMD. P10 fragments in the three receptors are highlighted with blue line. The replacement of FSHR residues to the correspond TSHR residues are labeled in yellow.

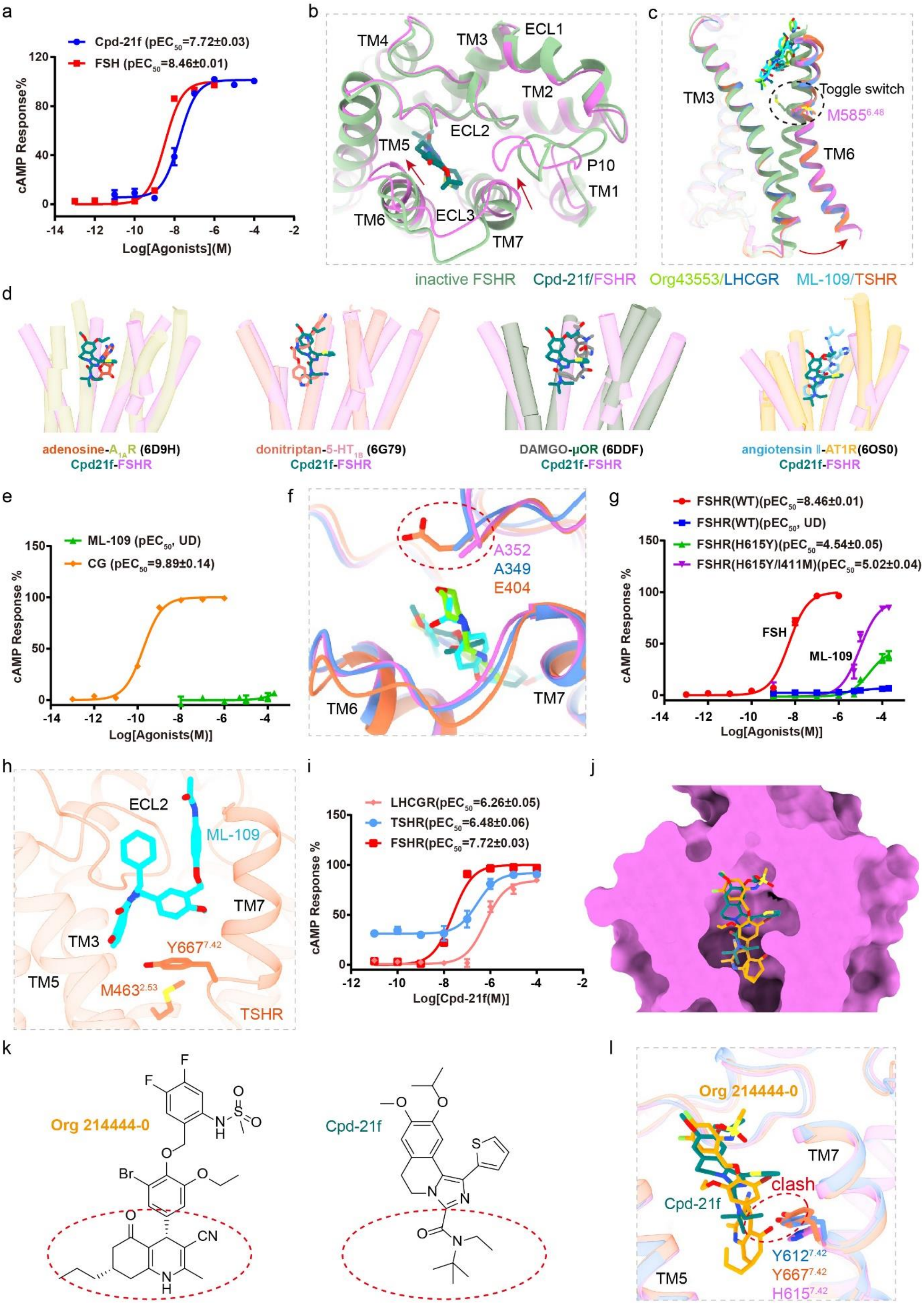

Supplementary Figure 7 | Allosteric agonists interact with glycoprotein hormone receptors.

a, Concentration-response curves for FSH and Cpd-21f-induced FSHR activation. The representative concentration-response curves from three independent experiments were shown.

b, Structural comparison of Cpd-21f bound active FSHR and inactive FSHR structures. The active FSHR is shown in violet and the inactive FSHR is shown in green. **c,** Structural comparison of the inactive FSHR, Cpd-2f bound FSH-FSHR-Gs complex, Org43553 bound CG-LHCGR-Gs complex, and ML-109 bound TSH-TSHR-Gs complex. **d,** Comparison the Cpd-21f binding pocket of FSHR with other orthosteric agonist-binding pockets of class A GPCRs. **e,** Concentration-response curves for CG and ML-109-induced LHCGR activation. The representative concentration-response curves from three independent experiments were shown. **f,** Structural comparison of the binding pocket in the FSH-FSHR-Gs complex with Org43553 binding pocket in the CG-LHCGR-Gs complex, and ML-109 binding pocket in the TSH-TSHR-Gs complex. A352 in FSHR, A349 in LHCGR and E404 in TSHR are shown in sticks. **g,** Concentration-response curves for WT and mutated FSHR induced by FSH and ML-109. The representative concentration-response curves from three independent experiments were shown. **h,** Residues $Y667^{7.42}$ and $M463^{2.53}$ in TSHR are located at the bottom of the ML-109 binding pocket. **i,** Concentration-response curves for Cpd-21f-induced FSHR, LHCGR and TSHR activation. The representative concentration-response curves from three independent experiments were shown. The data was normalized by FSHR response within each individual experiment, with the basal activity for FSHR as 0, while the fitted E_{max} of FSHR as 100. **j,** Comparison of the binding sites of Cpd-21f and org 214444-0 in the active FSHR structure. **k,** 2D structures of org214444-0 and Cpd-21f. The bottom hexahydroquinoline group of Org-214444-0 and the N-tert-Butyl-N-ethyl moiety of Cpd-21f are highlighted with red circles. **l,** Detailed structural comparison of the binding pockets in FSHR, LHCGR and TSHR. The clashes between org 214444-0 and residue $Y612^{7.42}$ of LHCGR and $Y667^{7.42}$ of TSHR are highlighted in red circle. Data were shown as mean ± S.E.M. from three independent experiments. Source data are provided with this paper.

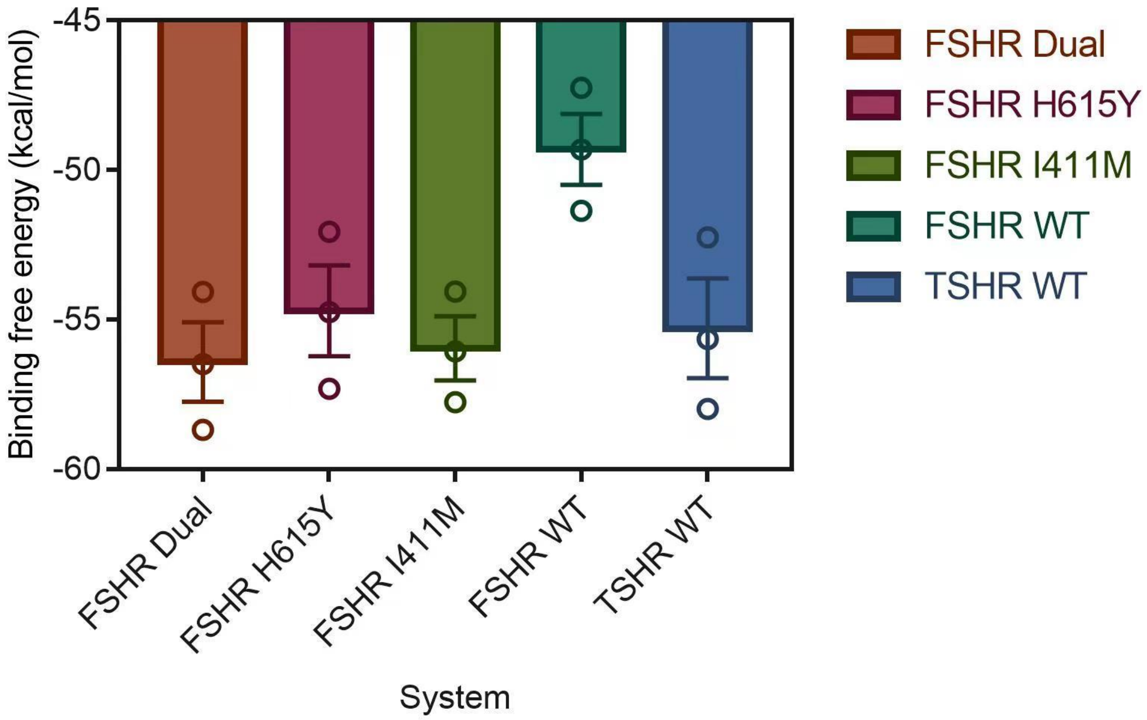

Supplementary Figure 8 | The binding free energy between ML-109 and the receptor calculated by MMGBSA. FSHR Dual means FSHR containing H615Y and I411M two mutations. The five MD simulation systems: FSHR(H615Y/I411M)-ML-109 (FSHR Dual), FSHR(H615Y)-ML-109, FSHR(I411M)-ML-109, FSHR(WT)-ML-109, and TSHR(WT)-ML-109. The 200 ns × 3 MD simulations were done, and the molecular mechanics energies combined with the generalized Born and surface area continuum solvation (MMGBSA) algorithm were used to calculate the binding free energy of ML-109 in each system. The last 1/4 of trajectories were used to guarantee that the trajectory had reached equilibrium upon evaluation. Data were shown as mean ± S.E.M. from three independent experiments. Source data are provided with this paper.

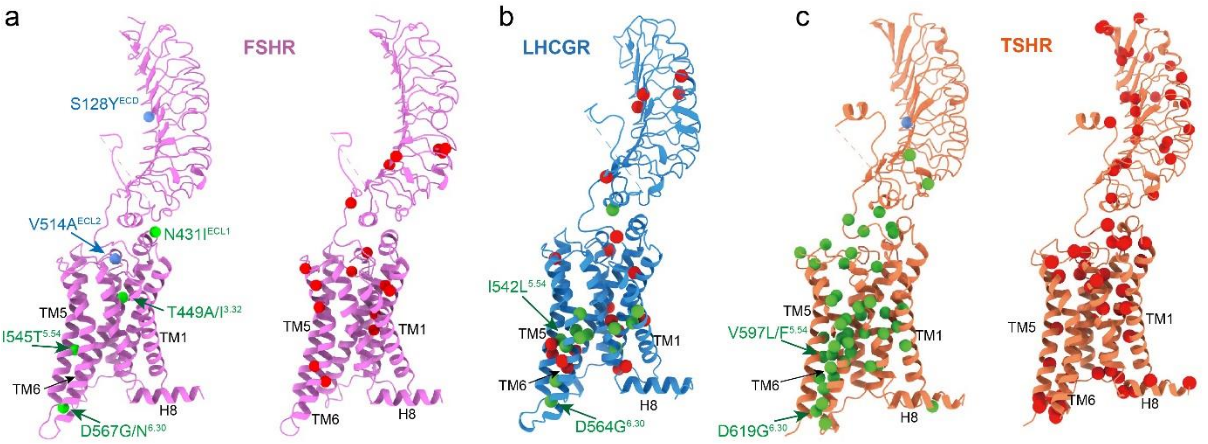

Supplementary Figure 9 | Distribution of diseases associated mutations on the FSHR, LHCGR and TSHR structures. a-c, The activating and inactivating point mutations in FSHR (**a**), LHCGR (**b**) and TSHR (**c**). The activating mutations are highlighted in green spheres, and inactivating mutations are shown in red sphere. FSHR with S128Y mutation and TSHR with K183R mutation show high sensitivity to hCG, S128 and K183 are highlighted in blue sphere. FSHR with V514A mutation results in higher level of surface expression, V514 is also highlighted in blue sphere.

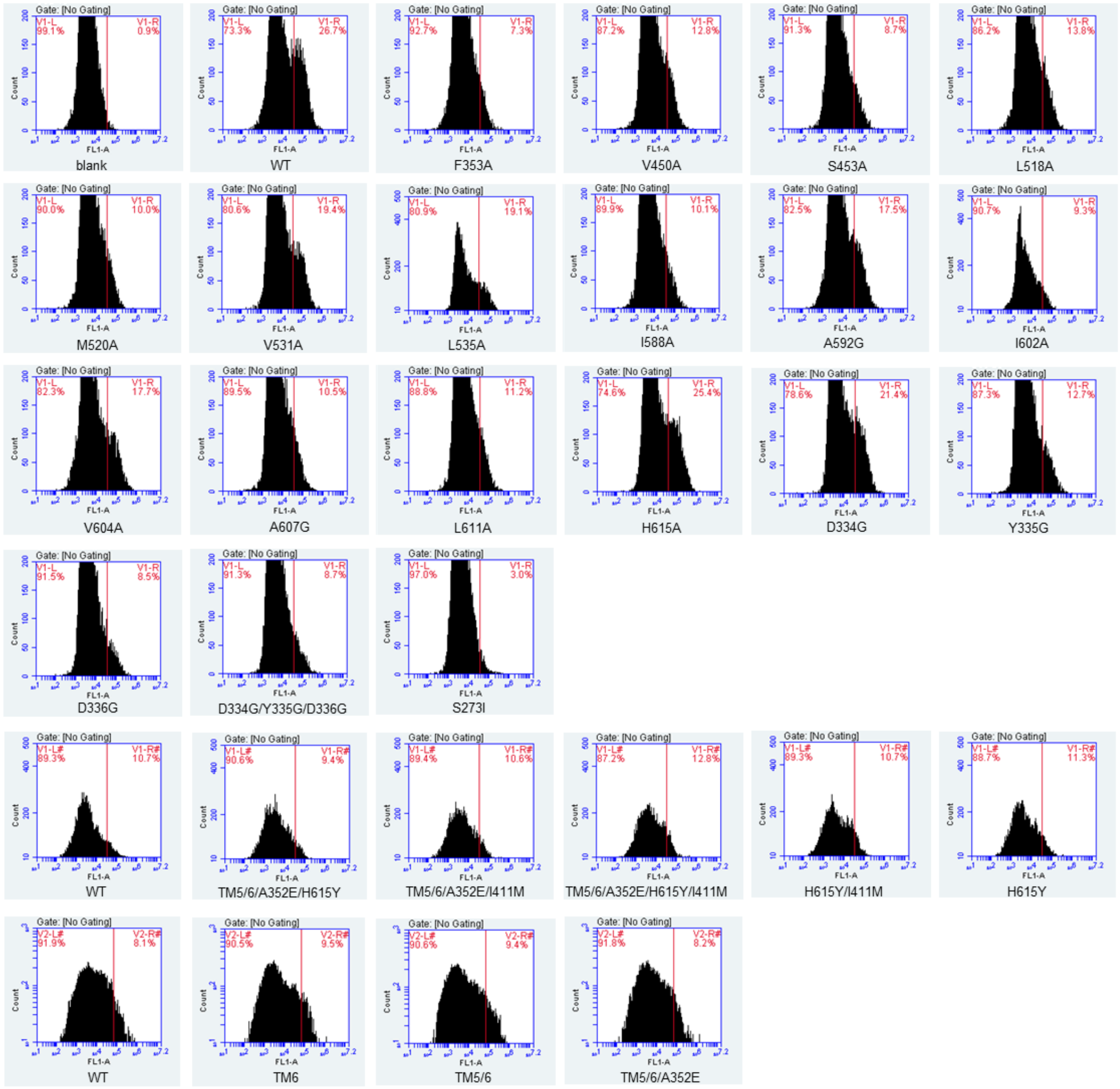

Supplementary Figure 10 | The representative flow cytometry data for the detection of receptor surface expression.

The fluorescence signal histogram map is divided into two regions according to the blank cell. The left and right regions represent the negative (FITC-) and positive cell communities (FITC+), respectively. The cell surface expression of WT FSHR and mutants can be calculated as follows: (M(FITC+)-M(FITC-)) × (FITC+% Parent). M, mean fluorescence intensity. Approximately 10,000 cellular events per sample were collected and data were finally normalized to WT FSHR.

Supplementary Table 1 | Cryo-EM data collection, refinement and validation statistics.

	FSH-FSHR-Gs	FSH-FSHR-ECD	FSHR
	EMD-35135		EMDB-35136
	PDB 8I2G		PDB 8I2H
**Data collection and processing**			
**Magnification**	48,543		46,685
**Voltage (kV)**	300		300
**Electron exposure (e–/Å^2)**	50		70
**Defocus range (μm)**	-0.8 to -2.0		-1.2 to -2.2
**Pixel size (Å)**	1.03		1.071
**Symmetry imposed**	C1		C1
**Initial particle images (no.)**	6,759,790		11,847,100
**Final particle images (no.)**	477,950		356,211
**Map resolution (Å)**	2.82	3.14	6.01
**FSC threshold**	0.143		0.143
**Map resolution range (Å)**	2.5 - 5.0		4.0-7.0
**Refinement**			
**Initial model used (PDB code)**	7FIH		FSH-FSHR-Gs
**Map sharpening *B* factor (Å^2)**	-142.2		
**Model composition**			
**Non-hydrogen atoms**	12,491		4,037
**Protein residues**	1,540		586
**Ligands**	1		0
**Lipids**	0		0
***B* factors (Å^2)**			
**Protein**	101.66		272.61
**Ligand**	86.16		-
**R.m.s. deviations**			
**Bond lengths (Å)**	0.003		0.02
**Bond angles (°)**	0.652		0.512
**Validation**			
**MolProbity score**	1.56		1.13
**Clashscore**	6.25		1.95
**Poor rotamers (%)**	0.52		0
**Ramachandran plot**			
**Favored (%)**	96.64		97.02
**Allowed (%)**	3.36		2.98
**Disallowed (%)**	0		0

Supplementary Table 2 | FSH and Cpd-21f-induced activation on wild type and FSHR with site-directed mutations.

Ligands	Constructs	pEC_{50}	E_{max}	Surface expression (%WT)
FSH	WT	8.46±0.01	100	100
	D334G	7.73±0.04***	97.02±1.04	76.24±2.11
	Y335G	7.22±0.01***	100.14±1.28	43.76±1.26
	D336G	8.38±0.08	95.02±0.69	29.62±0.35
	D334G/Y335G/D336G	5.95±0.04***	*UD*	28.48±0.97
	S273I	8.21±0.09**	93.00±1.87	41.24±2.84
Cpd-21f	WT	7.72±0.03	100	100
	F353A	8.36±0.05***	89.53±3.37	18.38±0.48
	V450A	6.31±0.04***	90.13±3.07	35.98±1.29
	S453A	7.10±0.02***	90.68±3.57	24.75±0.83
	L518A	6.93±0.04***	91.50±1.74	39.98±0.30
	M520A	8.16±0.05***	95.71±2.06	26.44±1.41
	V531A	7.41±0.02***	99.14±0.49	70.75±2.34
	L535A	7.14±0.04***	98.01±2.47	112.38±9.29
	I588A	7.26±0.06***	91.36±1.99	29.44±0.71
	A592G	6.46±0.03***	101.24±1.18	59.46±2.09
	I602A	7.11±0.09***	94.30±0.73	33.00±5.17
	V604A	7.31±0.02***	92.64±2.61	77.87±5.59
	A607G	7.02±0.01***	95.33±0.81	27.57±0.81
	L611A	7.29±0.03***	42.87±2.27	31.99±0.29
	H615A	6.83±0.06***	93.35±0.69	119.79±5.23
	H615Y	7.12±0.05***	95.06±0.38	88.21±5.81

Data were analyzed using ANOVA, and Graphpad Prism8.0, the three-parameter, nonlinear regression equation in Prism suite was used in fitting. Data were represented as mean pEC_{50} (pEC_{50} ± SEM). Experiments were performed at least three times, each experiment conducted in triplicate. **P<0.01, ***P<0.001 versus WT. *UD*, undetectable. Statistical significance of differences between WT and mutants was determined by two-sided one-way ANOVA with Tukey test. Source data are provided with this paper.

Supplementary Table 3 | Allosteric agonists-induced activation on wild type and mutated FSHR.

Ligands	Constructs	pEC_{50}	E_{max}	Surface expression (%WT)
FSH	WT	8.46±0.01	100	100
ML-109	WT	*UD*	*UD*	100
	TM6	*UD*	*UD*	77.47±11.42
	TM5/TM6	*UD*	*UD*	89.95±8.47
	TM5/TM6/A352E	*UD*	*UD*	75.39±12.68
	TM5/TM6/A352E/H615Y	5.98±0.01	87.61±0.34	70.80±3.01
	TM5/TM6/A352E/I411M	4.95±0.05	92.71±1.07	94.96±2.77
	TM5/TM6/A352E/H615Y/I411M	6.24±0.01	99.29±0.22	117.00±18.31
	H615Y	4.54±0.05	45.24±2.94	88.21±5.81
	H615Y/I411M	5.02±0.04	88.74±1.66	97.01±4.83

Data were analyzed using Graphpad Prism8.0, the three-parameter, nonlinear regression equation in Prism suite was used in fitting. Data were represented as mean pEC_{50} (pEC_{50} ± SEM). Experiments were performed at least three times, each experiment conducted in triplicate. Constructs TM6 means swapping of the extracellular portion of FSHR TM6 (residues 591-599) with the corresponding TSHR TM6 region (residues 643-651); TM5/TM6 means the replacement of the extracellular portions of both TM5 and TM6 from FSHR (residues 527-536/591-599) with the corresponding TSHR TM5/TM6 regions (residues 579-588 and 643-651); TM5/TM6/A352E means mutation of A352E in addition to the above TM5/TM6 mutations; TM5/TM6/A352E/H615Y means mutation of $H615^{7.42}Y$ in addition to the above TM5/TM6/A352E mutations; TM5/TM6/A352E/I411M means mutation of $I411^{2.53}M$ in addition to the above TM5/TM6/A352E mutations; TM5/TM6/A352E/H615Y/I411M means mutations of $H615^{7.42}Y$ and $I411^{2.53}M$ in addition to the above TM5/TM6/A352E mutations. *UD*, undetectable. Source data are provided with this paper.

.

Supplementary Table 4 | Agonists-induced activation on wild type LHCGR and TSHR.

Ligands	Receptor	pEC_{50}
CG	LHCGR	9.89±0.14
ML-109		*UD*
Cpd-21f		6.26±0.05
	TSHR	6.48±0.06

Data were analyzed using Graphpad Prism8.0, the three-parameter, nonlinear regression equation in Prism suite was used in fitting. Data were represented as mean pEC_{50} (pEC_{50} ± SEM). Experiments were performed at least three times, each experiment conducted in triplicate. *UD*, undetectable. Source data are provided with this paper.
