# Supplementary material for: Mechanism of hormone and allosteric agonist mediated activation of follicle stimulating hormone receptor
Source: Nat Commun. 2023 Jan 31;14:519. doi: 10.1038/s41467-023-36170-3 (PMC9889800; doi:10.1038/s41467-023-36170-3)
Supplement: Supplementary file 3 — Reporting Summary [file 41467_2023_36170_MOESM3_ESM.pdf]

nature portfolio

Corresponding author(s): H. Eric Xu
Last updated by author(s): Jan 14, 2023

Reporting Summary

Nature Portfolio wishes to improve the reproducibility of the work that we publish. This form provides structure for consistency and transparency in reporting. For further information on Nature Portfolio policies, see our Editorial Policies and the Editorial Policy Checklist.

Statistics

For all statistical analyses, confirm that the following items are present in the figure legend, table legend, main text, or Methods section.

n/a	Confirmed	
☐	☒	The exact sample size (*n*) for each experimental group/condition, given as a discrete number and unit of measurement
☐	☒	A statement on whether measurements were taken from distinct samples or whether the same sample was measured repeatedly
☐	☒	The statistical test(s) used AND whether they are one- or two-sided<br>*Only common tests should be described solely by name; describe more complex techniques in the Methods section.*
☒	☐	A description of all covariates tested
☒	☐	A description of any assumptions or corrections, such as tests of normality and adjustment for multiple comparisons
☐	☒	A full description of the statistical parameters including central tendency (e.g. means) or other basic estimates (e.g. regression coefficient) AND variation (e.g. standard deviation) or associated estimates of uncertainty (e.g. confidence intervals)
☐	☒	For null hypothesis testing, the test statistic (e.g. F, t, r) with confidence intervals, effect sizes, degrees of freedom and P value noted<br>*Give P values as exact values whenever suitable.*
☒	☐	For Bayesian analysis, information on the choice of priors and Markov chain Monte Carlo settings
☒	☐	For hierarchical and complex designs, identification of the appropriate level for tests and full reporting of outcomes
☒	☐	Estimates of effect sizes (e.g. Cohen's *d*, Pearson's *r*), indicating how they were calculated

Our web collection on statistics for biologists contains articles on many of the points above.

Software and code

Policy information about availability of computer code

Data collection	Cyro-EM data were automatically collected using serialEM 3.7 and EPU 2.12 on a Titan Krios.
Data analysis	The following softwares were used in this study: MotionCor 2.1, gCTF v1.18, RELION-3.1.2, CryoSPARC-v3.3, UCSF Chimera 1.14, UCSF ChimeraX 1.3, Phenix 1.18, Coot 0.9.4.1, ISOLDE 1.2, Graphpad Prism 8.0, Pymol 2.0.3.0, MolProbity 4.2, BD Accuri C6 software 1.0.264.21.

For manuscripts utilizing custom algorithms or software that are central to the research but not yet described in published literature, software must be made available to editors and reviewers. We strongly encourage code deposition in a community repository (e.g. GitHub). See the Nature Portfolio guidelines for submitting code & software for further information.

Data

Policy information about availability of data

All manuscripts must include a data availability statement. This statement should provide the following information, where applicable:

- Accession codes, unique identifiers, or web links for publicly available datasets
- A description of any restrictions on data availability
- For clinical datasets or third party data, please ensure that the statement adheres to our policy

The density maps and structure coordinates have been deposited to the Electron Microscopy Database (EMDB) and the Protein Data Bank (PDB) with accession number of EMD-35135, PDB ID 8I2G for the FSH-FSHR-Gs complex; EMD-35136 and 8I2H for the inactive FSHR.

Human research participants

Policy information about studies involving human research participants and Sex and Gender in Research.

Reporting on sex and gender	*Use the terms sex (biological attribute) and gender (shaped by social and cultural circumstances) carefully in order to avoid confusing both terms. Indicate if findings apply to only one sex or gender; describe whether sex and gender were considered in study design whether sex and/or gender was determined based on self-reporting or assigned and methods used. Provide in the source data disaggregated sex and gender data where this information has been collected, and consent has been obtained for sharing of individual-level data; provide overall numbers in this Reporting Summary. Please state if this information has not been collected. Report sex- and gender-based analyses where performed, justify reasons for lack of sex- and gender-based analysis.*
Population characteristics	*Describe the covariate-relevant population characteristics of the human research participants (e.g. age, genotypic information, past and current diagnosis and treatment categories). If you filled out the behavioural & social sciences study design questions and have nothing to add here, write "See above."*
Recruitment	*Describe how participants were recruited. Outline any potential self-selection bias or other biases that may be present and how these are likely to impact results.*
Ethics oversight	*Identify the organization(s) that approved the study protocol.*

Note that full information on the approval of the study protocol must also be provided in the manuscript.

Field-specific reporting

Please select the one below that is the best fit for your research. If you are not sure, read the appropriate sections before making your selection.

☒ Life sciences ☐ Behavioural & social sciences ☐ Ecological, evolutionary & environmental sciences

For a reference copy of the document with all sections, see nature.com/documents/nr-reporting-summary-flat.pdf

Life sciences study design

All studies must disclose on these points even when the disclosure is negative.

Sample size	For cryo-EM data, images were collected until the resolution and 3D reconstruction converges.<br>For all the functional assay, no statistical approaches were used to predetermine the sample size. We use sample size at least of three independent experiments, commonly exploited by researchers in this field.<br>For structural determination, 4495 movies of the inactive FSHR protein, 4025 movies for the FSH-FSHR-Gs complex were collected using an Titan Krios equipped with a Gatan K3 Summit direct electron detector.<br>For cAMP accumulation, three biologically independent experiments (n=3) were performed as depicted in related Figure legends. Data were analysed by fitting various ligand concentrations and readouts using appropriate equations in GraphPad Prism 8.0.
Data exclusions	No data were systematically excluded from the analysis except badly picked or low resolution particles from cryo-EM data.
Replication	Each experiment was reproduced at least three times independently. Experimental findings were reliably reproduced.
Randomization	No Randomization was attempted or needed. Randomization was not necessary as the independent variables to be tested were sufficient for the functional interpretation within this study. i.e. WT vs mutant vs control conditions or dose-response determination.
Blinding	Cryo-EM data were then collected automatically. For functional analysis, blinding was not necessary due to the quantitative nature of the experiment. All experimental data acquired or analyzed in this study are included in this published article, and subjected to statistical analysis whenever necessary.

Reporting for specific materials, systems and methods

We require information from authors about some types of materials, experimental systems and methods used in many studies. Here, indicate whether each material, system or method listed is relevant to your study. If you are not sure if a list item applies to your research, read the appropriate section before selecting a response.

Materials & experimental systems

n/a	Involved in the study
☐	☒ Antibodies
☐	☒ Eukaryotic cell lines
☒	☐ Palaeontology and archaeology
☒	☐ Animals and other organisms
☒	☐ Clinical data
☒	☐ Dual use research of concern

Methods

n/a	Involved in the study
☒	☐ ChIP-seq
☐	☒ Flow cytometry
☒	☐ MRI-based neuroimaging

Antibodies

Antibodies used

For measurement of receptor cell surface expression, Mouse monoclonal anti-FLAG M2 antibody (Abclonal, AE005) and goat anti-mouse IgG(H+L) Highly Cross-Adsorbed Secondary Antibody, Alexa Fluor 488 (Invitrogen, NO.A-11029) were used. The primary antibody was used in 1:150 dilution, and the secondary antibody in 1:1000 dilution.

Validation

Mouse anti DDDDK-Tag mAb: https://abclonal.com/catalog-antibodies/MouseantiDDDDKTagmAb/AE005
Goat anti-Mouse IgG (H+L) Highly Cross-Adsorbed Secondary Antibody, Alexa Fluor 488: https://www.thermofisher.com/cn/en/antibody/product/Goat-anti-Mouse-IgG-H-L-Highly-Cross-Adsorbed-Secondary-Antibody-Polyclonal/A-11029
All antibodies used are commercially purchased and have been validated by the vendors. Validation data are available from the respective vendor's respective websites.

Eukaryotic cell lines

Policy information about cell lines and Sex and Gender in Research

Cell line source(s)

Mammalian CHO-K1 cells: ATCC no. CCL-61, Insect cells: Invitrogen (Sf9)

Authentication

All of the cell lines are maintained by the supplier. No additional authentication was performed by the authors of this study.

Mycoplasma contamination

The above cell lines were negative for Mycoplasma contamination.

Commonly misidentified lines
(See ICLAC register)

No commonly misidentified cell lines were used.

Flow Cytometry

Plots

Confirm that:

☒ The axis labels state the marker and fluorochrome used (e.g. CD4-FITC).

☒ The axis scales are clearly visible. Include numbers along axes only for bottom left plot of group (a 'group' is an analysis of identical markers).

☒ All plots are contour plots with outliers or pseudocolor plots.

☒ A numerical value for number of cells or percentage (with statistics) is provided.

Methodology

Sample preparation

CHO-K1 cells (ATCC, #CCL-61) were cultured in Ham's F-12 Nutrient Mix (Gibco) supplemented with 10% (w/v) fetal bovine serum. Cells were maintained at 37°C in a 5% CO2 incubator with 150,000 cells per well in a 12-well plate. Cells were grown overnight and then transfected with 1μg FSHR (or LHCGR, or TSHR) constructs by FuGENE® HD transfection reagent in each well. After 24h of transfection, cells were washed once with PBS and then detached with 0.2% (w/v) EDTA in PBS. Cells were blocked with PBS containing 5% (w/v) BSA for 15min at room temperature (RT) before incubating with primary anti-Flag antibody (diluted with PBS containing 5% BSA at a ratio of 1:150, Abclonal) for 1h at RT. Cells were then washed three times with PBS containing 1% (w/v) BSA and then incubated with anti-mouse Alexa-488-conjugated secondary antibody (diluted at a ratio of 1:1000, Invitrogen) at 4°C in the dark for 1h. After another three times of wash, cells were harvested, and fluorescence intensity was quantified in a BD Accuri C6 flow cytometer system (BD Biosciences)

Instrument

BD Accuri C6 (BD Biosciences)

Software

BD Accuri C6 software (version 1.0.264.21)

Cell population abundance

Approximately 10,000 cellular events were collected and the total fluorescence intensity of positive expression cell population was calculated.

Gating strategy

Gating was determined by the Alexa-488 fluorescence intensity to differentiate positive cells and all other cells.

☒ Tick this box to confirm that a figure exemplifying the gating strategy is provided in the Supplementary Information.
